# Supplementary material for: Patterns of Intron Gain and Loss in Fungi
Source: PLoS Biol. 2004 Nov 30;2(12):e422. doi: 10.1371/journal.pbio.0020422 (PMC532390; doi:10.1371/journal.pbio.0020422)
Supplement: Table S1 — Also available at http://genes.mit.edu/NielsenEtAl/. (4.3 MB ZIP). [file pbio.0020422.st001.zip › NielsenEtAl/html/877.html]

AN1900.1.NCU04713.1.MG02501.1.FG09462.1


```
 CLUSTAL W (1.82) Multiple Sequence Alignments - Introns Inserted


Sequence 1: NCU04713.1	728 aa
Sequence 2: MG02501.1	694 aa
Sequence 3: FG09462.1	687 aa
Sequence 4: AN1900.1	710 aa
Alignment Length: 759 aa
Number Identitical Residues: 188 aa
Alignment Score (without introns) 12086


MG02501.1 	-----------------MSTTDRGASGTDSQQAVAEVSSSRVVPKPVPQSQARDTRAYQL
NCU04713.1	MIPIRPKPNGNNPDGGGSSTTTTSPPPRRDKQAVHTPSPSRITSKPVPERQLRSPREYQI
FG09462.1 	----------------MISTNSNPDSPQTRKQVVAEASSSRVLPKPVPESQAQDPRTYQI
AN1900.1  	-----------------MSGSTTP-----------KTTTTKASQRPISRTESSNPRAFQI
          	                  * .                :.::   :*:.. :  ..* :*:

MG02501.1 	QQLRKRYSP-KELSPNGEGGVAYLQFRMKPSDPDFPYELDHLEVELRIPSTYPKE-PPSL
NCU04713.1	DQLKRRFSPLKETIDPSTGGETSLLFSLKPSDPDFPYDLEKLECDLRIPGEYPRQ-PARL
FG09462.1 	DQLRRRYSP-KETTAP--DGATSLAFKMKPSDPDFPFELSTLECDIRVPQAYPDE-TPVL
AN1900.1  	NQLRRRFRP----TESNDESGTTLSFELIPSDPDFPFELERLQCALYVPLTYPARGRPSL
          	:**::*: *       .  . : * * : *******::*. *:  : :*  ** .. . *

MG02501.1 	SVENRDIPRGFRINIERGWDKLVAE--RKG--ASTLLSLMNALDRNLETFLSEQKAETVK
NCU04713.1	LVRNKDIPRGFAVNIERGWDRLVEE--RKGKGTTTLLSVLNALDRGLEGFLAEEKKETVK
FG09462.1 	HVKNKDIPRGFSINVERGWDSLVEE--KPG---ATLLAYTRELDKNLEKFLSEQKAETVK
AN1900.1  	KVANPEIGAAFQENISRGFDDIVDSSLRMG-GRGTLLNYMNSLDRHLERLLTTTER-GPT
          	 * * :*  .*  *:.**:* :* .: : * .  ***   . **: ** :*:  :    .

MG02501.1 	MTIFKDTRHLDAMAA--------EAASQPETVVQ-PPPQR-----RPLYRPETFTREQVT
NCU04713.1	ITVFKDSRHLEGQPSGVIGKEGIEGVSKEKNENE-KEKEKEKEKPRPYIPPENFTRDQIA
FG09462.1 	LVTFRDTRHVEAQKAQIQSQSLTQESAQPKKPSE-PAPPKPAPPPVRYVPEPSYTKEETT
AN1900.1  	LKFVQNARKEESSKTLVDNMNSLALAQGAESRRDGSPAPVTAPSPAPAEVRPVYTAEERA
          	:  .:::*: :.  :   . .        :.  :.         .        :* :: :

MG02501.1 	EAKAIRAQETRQLEARMGRLPLYQKSSDGIVYTLPLDPKRRSELPVGLRPVKSVQLIIPL
NCU04713.1	EAKARRAQEVRQLEARMSGLPLYHKSSDGVIYTLPLDPKRRSSLPTGLQSVHSVQLVIPL
FG09462.1 	AAKERRASEIRQLEARMKRTSEYRRSPDGVVYTLPLEPQKRDQLPPGLQSVRSLHLIIPF
AN1900.1  	QAERRRAVETKQLEARLQRLPLFQKARDSLSFVIPVQPTKVDRLPIALRPVKTVKLSVPL
          	 *:  ** * :*****:   . :::: *.: :.:*::* : . ** .*:.*::::* :*:

MG02501.1 	LYPLQQIRVLLNDVASDEAEPVEEIFAEKAAAQRTMNLMSQVNYLTQNIHVLARQVQANR
NCU04713.1	LYPLQPLRVLLNDVDADEAETVEELFTQKAVEQKQMSLTSHLNYLATNLHALAKQAAQQK
FG09462.1 	LYPLQNLRIQLNNVEPQDAEPVEELFCEKAAEQQHMTLMSHLNYLAQNIHKLAKQAQAQ-
AN1900.1  	LYPLEPSSIEIQGIHGNEARAVEAGFSQWLKDHLHLNLVSQVNHLASNIHNFAKTVVTEP
          	****:   : ::.:  ::*..**  * :    :  :.* *::*:*: *:* :*: .  : 

MG02501.1 	EAALAAERMQAKVERKAEDVDGHGKAAAHVS-SLDDVKGHVKVIPRPPEWSYGNA-GESE
NCU04713.1	QKAVQAA-AAAEEEGKKMAADEEAKEAEHVASAVDGERGHVHVIPRPLEWTLVNADGSSE
FG09462.1 	---------APKIETPAPAAEDQSQDTKEPE--PKSEKSHIQVIPRPPEWGYVDA-----
AN1900.1  	D--------EPKQPELETKVLEEPQPPVEDHSGTNEDKPHIHVIQRPPEWTVSNG-----
          	.         .:       .  . : . .  :. .  : *::** ** **   :.     

MG02501.1 	SDSEDSYDSDEDGQGGASLGPLDAQEPTSSSTRMTEKGTAITFPSIELYGVEILEINTLS
NCU04713.1	SESETDSDDDYELVDGESGNASGVTPGTALPTATAERGTAISFPSIELHGIELLQVTLLS
FG09462.1 	-DDEESSSSDEADEGGAAVEDEEKEPQVYMPGETPERGTMVSFPSIELHGIELLQLAILG
AN1900.1  	ASGSEETDITTTEDDCSDDETDGGAPVPDLPEPATEHGVALSFPFLELYGIELLELVGLY
          	:... . .      .               .   .*:*. ::** :**:*:*:*::  * 

MG02501.1 	VSVKCLRCKTNNDLGGLKHN-------VDKVSSCKKCATEFVAT~FRPELVHQNSTRAGF
NCU04713.1	LSVKCNRCKTLNDVTSLKSN-------VEKLASCKKCATSFTVR~FRAELVHANSTRAGF
FG09462.1 	ISIKCERCKTLNEVTGLKPD-------VEKTTSCKKCATAFAVK~FRQTLIHMNSTRAGF
AN1900.1  	ITVKCDRCKEQLDVRNIPQTKDSSGVLSPKVEVCKKCTNPMSLG1FRRLLMHPNSNRAGY
          	:::** ***   :: .:   ..::.    *   ****:. :    **  *:* **.***:

MG02501.1 	INLSGCTVTDLLPS~TFVPTCSKCST-AAAQGFVSVRGDATTNVCRECHGRFT~FKIPEV
NCU04713.1	IDLSGCTISDMLPS~SFIPTCGRCSTPYPSPGFVSVRGESTTNVCRECHARFT~FKLPDV
FG09462.1 	LDLAGCKVADMLPS~TFVPTCERCST--PAPGLVSVRGSSVTNVCRDCHGHFT~FKIPEV
AN1900.1  	LDLDGCTVVDLLPS2GFIPTCAECSTPFPGPGVVAVRGESAMANCRQCHRKLV1FKIPEV
          	::* **.: *:***  *:*** .***. .. *.*:***.:.   **:** ::. **:*:*

MG02501.1 	KLQSYSSGAS~GSLPPTSGPRR~RQEKLGLHAGDPLPARGACEHYKKSYRWFR-------
NCU04713.1	KFLTYSSSQS~-HLPPTQGPKR~KDQKLGLHAGDPLPAKGACAHYKRSYRWFR-------
FG09462.1 	KFLFITPGSR~--PPPTSGPRR~KQEKLGLHAGDQLPDKGTCSHYRKSYRWFR-------
AN1900.1  	KFLKVGSAAF1TSRERAPARKK0PKEVLGIVAGQELPRRGRCMHYSKSYRWFRYLIYFSS
          	*:    ..        : . ::  .: **: **: ** :* * ** :******     ::

MG02501.1 	----FSCCDRVSACDKCHDAEA~DGHVHEWANRMIC~GWCSREQNYR--VDSCGFCGRSV
NCU04713.1	----FSCCNKVYPCDKCHQQAE~D-HVDEWANRMIC~GWCSREQNYR--VENCAFCGRSV
FG09462.1 	----FSCCSRVHPCDKCHDKQE~D-HINEWANRIIC~GWCSREQNYSSAVEACRFCGRFL
AN1900.1  	YMAVFLHLTDSAAVRKSFLVTT1TDHPNEHANRMIC2GFCSREQIYR--PENCGICRSYL
          	  : *       .  *..      .* .* ***:** *:***** *    : * :*   :

MG02501.1 	VGRRGTGYWEGGKGTRDQVLMRRND~KRKHRR----IG~GGEGKKKE-
NCU04713.1	VRKRTGGFWEGGKGTRDKVLMRRGD~RRKYRN----KK~DGNKSNKEK
FG09462.1 	IGKKGRGFWEGGKGTRDRTTMSRKD~PRKYKR----IV1WESRFLSA-
AN1900.1  	IGKAGSGFWEGGKGTRNKTLMSRKD1PRKYKRRPGSTP~GGTSSSKKK
          	: :   *:********::. * * *  **::. ..:         . .
```
